# COL10A1 Overexpression Promotes Gastric Cancer Aggressiveness Through EMT and Major Oncogenic Pathways

**DOI:** 10.3390/ijms262211043

**Published:** 2025-11-14

**Authors:** Laura G. Necula, Denisa L. Dragu, Lilia Matei, Ioana Pitica, Simona O. Dima, Coralia Bleotu, Carmen C. Diaconu, Mihaela Chivu-Economescu

**Affiliations:** 1 Cellular and Molecular Pathology Department, Stefan S. Nicolau Institute of Virology, 030304 Bucharest, Romania; denisa.dragu@virology.ro (D.L.D.); lilia.matei@virology.ro (L.M.); ioana.pitica@virology.ro (I.P.); coralia.bleotu@virology.ro (C.B.); carmen.diaconu@virology.ro (C.C.D.); 2 Faculty of Medicine, Titu Maiorescu University, 040441 Bucharest, Romania; 3 Center of Excellence in Translational Medicine, Fundeni Clinical Institute, 020021 Bucharest, Romania; dima.simona@gmail.com; 4 Center of Digestive Diseases and Liver Transplantation, Fundeni Clinical Institute, 020021 Bucharest, Romania; 5 Faculty of Medicine, Carol Davila University of Medicine and Pharmacy, 050474 Bucharest, Romania; 6 Department of Strategy, Education, Documentation, and IT (SEDI), Stefan S. Nicolau Institute of Virology, 030304 Bucharest, Romania; mihaela.economescu@virology.ro

**Keywords:** COL10A1, collagen, gastric cancer, tumor microenvironment

## Abstract

Gastric cancer (GC) remains a major cause of cancer-related mortality, with limited options for early detection and precision therapy. Collagen family members are increasingly recognized as key structural and regulatory components of the tumor microenvironment. *Collagen type X alpha 1 chain* (*COL10A1*) appears among the top overexpressed genes in GC and has been linked with tumorigenesis, but its functional role in GC has not been completely elucidated. The oncogenic potential of COL10A1 was assessed in vitro in GC cell lines using adenoviral-mediated overexpression. Functional assays were further performed to evaluate proliferation, apoptosis, migration, invasion, and epithelial–mesenchymal transition (EMT) markers. Intracellular signaling alterations were analyzed by phosphokinase protein profiling and protein–protein interaction network analysis. *COL10A1* overexpression significantly increased proliferation and migration, while reducing GC cell apoptosis. It promotes EMT by up-regulating mesenchymal markers (*N-cadherin*, *Vimentin*, *Snail/Slug*) and suppressing epithelial markers such as *E-cadherin* and *β-catenin*. Additionally, *COL10A1* overexpression activated oncogenic signaling pathways, including the JNK and MAPK cascades, increasing proliferation and tumorigenic potential. Our results showed that COL10A1 functions as a driver for tumor progression by promoting proliferation, migration, and invasion along with EMT through activation of important oncogenic pathways. These findings highlight its biological role in tumor progression and contribute to a better understanding of GC pathogenesis.

## 1. Introduction

Despite advances in targeted cancer therapy and immunotherapy, GC still represents a major global health concern, being the fifth leading cause of cancer deaths, and the fifth most frequently diagnosed cancer [1]. The poor overall prognosis of GC is primarily attributed to diagnosis in advanced stages, resulting from the late onset of symptoms and the absence of reliable biomarkers with high specificity and sensitivity. The standard curative approach includes gastrectomy and lymphadenectomy, often enhanced by perioperative chemotherapy and radiotherapy [2].

The GC tumor microenvironment is a heterogeneous network of cancer cells, cancer-associated fibroblasts, collagen fibers, and other extracellular matrix components, such as immune cells, endothelial cells, and a complex network of cytokines, chemokines, and growth factors that interact to collectively promote tumor growth, invasion, metastasis, and resistance to therapy [3].

The collagen family represents the most abundant proteins in mammals, containing 28 members that are associated with the occurrence, progression, and prognosis of several cancer types, such as breast, colorectal, gastric, lung, and cervical carcinomas [4]. The involvement of collagens in carcinogenesis appears to occur within the tumor microenvironment, where these proteins form a structural framework that modulates immune responses, angiogenesis, and tumor progression [5].

Our previous studies showed that the collagen family and other proteins associated with the assembly mechanism of collagen fibers and with their degradation play an important role during the carcinogenesis process, and could be important biomarkers that predict poor prognosis in GC [6]. COL10A1 from tissue and its soluble form in plasma were previously identified by us in a series of studies as being associated with early GC and tumor progression [7].

This study aims to elucidate the role of COL10A1 in GC pathogenesis, focusing on the effects associated with its overexpression. Our study provides a detailed characterization of its functional consequences, as this alteration is predominant in the analyzed cohorts and in publicly available datasets (TCGA, GEO, etc.), and is significantly correlated with an aggressive tumor phenotype.

## 2. Results

### 2.1. COL10A1 Is Overexpressed in GC

**Figure 1:**
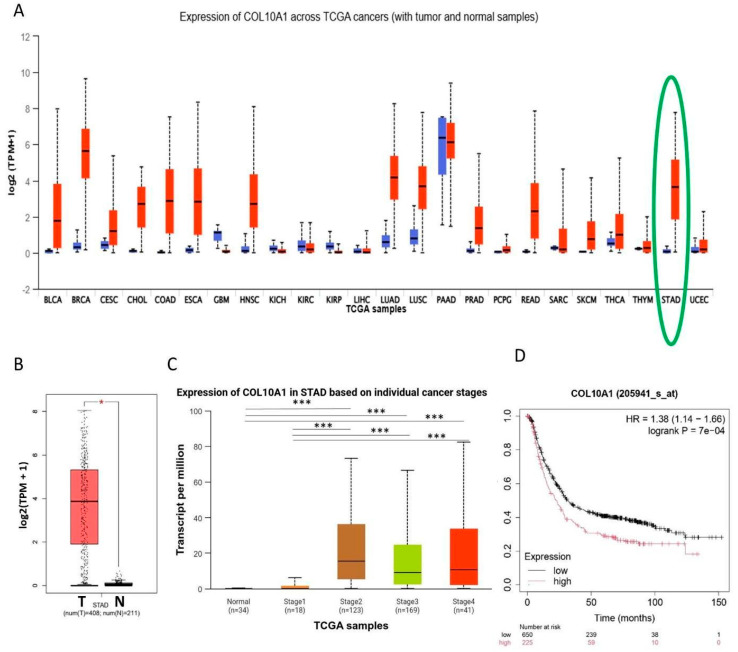
*COL10A1* is highly expressed in GC tissues and associated with OS. (**A**) High expression of *COL10A1* was detected in 18 solid tumors, including BLCA, BRCA, CESC, CHOL, COAD, ESCA, HNSC, LUAD, LUSC, PRAD, PCPG, READ, SARC, SKCM, THCA, THYM, STAD (highlighted in green), and UCEC, and low *COL10A1* expression was detected in 3 solid tumors, GBM, KICH, and KIRP. (**B**) *COL10A1* is upregulated in GC tissues (*n* = 408) compared with normal adjacent tissue (*n* = 211). (**C**) *COL10A1* is overexpressed in early (stage II) and advanced stages (III, IV) of GC. (**D**) Survival analysis using Kaplan–Meier Plotter. (* *p* < 0.05; *** *p* < 0.001).

Bioinformatic analysis of existing data showed that *COL10A1* is markedly overexpressed in multiple cancers, with particularly strong upregulation in GC, also known as stomach adenocarcinoma (STAD), compared to normal tissues (Figure 1A,B). Elevated expression is observed across all clinical stages of GC, suggesting its involvement throughout tumor progression (Figure 1C). Survival analysis further demonstrates that patients with high *COL10A1* expression have significantly poorer overall survival (Figure 1D). These results suggest that COL10A1 may serve as a prognostic biomarker and therapeutic target in GC.

### 2.2. Upregulation of COL10A1 Expression by Cell Transduction

**Figure 2:**
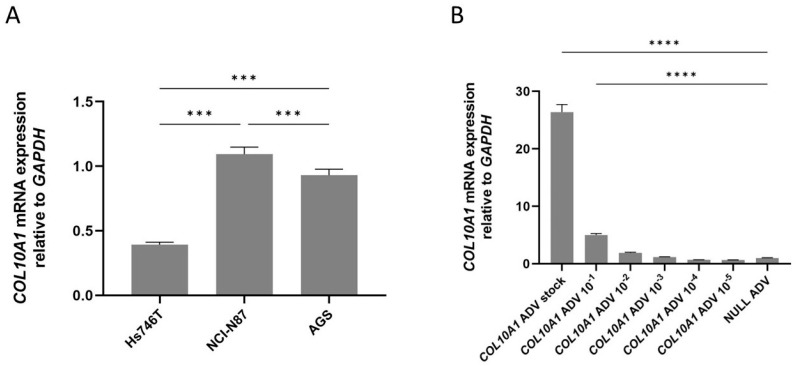
(**A**) *COL10A1* mRNA expression in GC cell lines Hs746T, NCI-N87, and AGS, normalized to the housekeeping gene *GAPDH*. (**B**) *COL10A1* gene expression in the GC cell line Hs746T following treatment with *COL10A1* ADV at different concentrations, normalized to the housekeeping gene *GAPDH*, 48 h post-transduction. *** (*p* < 0.001), **** (*p* < 0.0001).

To evaluate the effect of *COL10A1* overexpression on tumor processes, we first analyzed *COL10A1* expression in GC cell lines Hs746T, AGS, and NCI-N87. We selected these three gastric cell lines based on our experience with in vitro GC studies and because they represent distinct molecular subtypes of GC. Hs746T cells exhibit a mesenchymal phenotype with high invasive potential [8]; NCI-N87 cells display epithelial features and HER2 overexpression [9], while AGS cells represent a moderately differentiated, intestinal-type model [10]. Our investigation showed that *COL10A1* mRNA expression was significantly lower in the Hs746T cell line compared with NCI-N87 (*p* = 0.0004) and AGS (*p* = 0.0004) (Figure 2A), therefore we selected this line for subsequent transduction experiments. In the next step, we tested different concentrations of *COL10A1* ADV to optimize the cell transduction protocol. The results showed that the use of the *COL10A1* ADV system induced a 26-fold increase in *COL10A1* gene expression in the Hs746T cell line compared to the control cells treated with NULL ADV (*p* < 0.0001), at 48 h post-transduction, using *COL10A1* ADV stock concentration (Figure 2B). This transduction protocol was selected for the following experiments that analyzed the effects induced by *COL10A1* overexpression on the functionality of gastric tumor cells: evaluation of proliferative capacity, apoptosis, invasiveness capacity, EMT markers, and analysis of the main signaling pathways involved in gastric carcinogenesis.

### 2.3. COL10A1 Overexpression Promotes Gastric Cancer Cell Proliferation and Decreases Apoptosis

**Figure 3:**
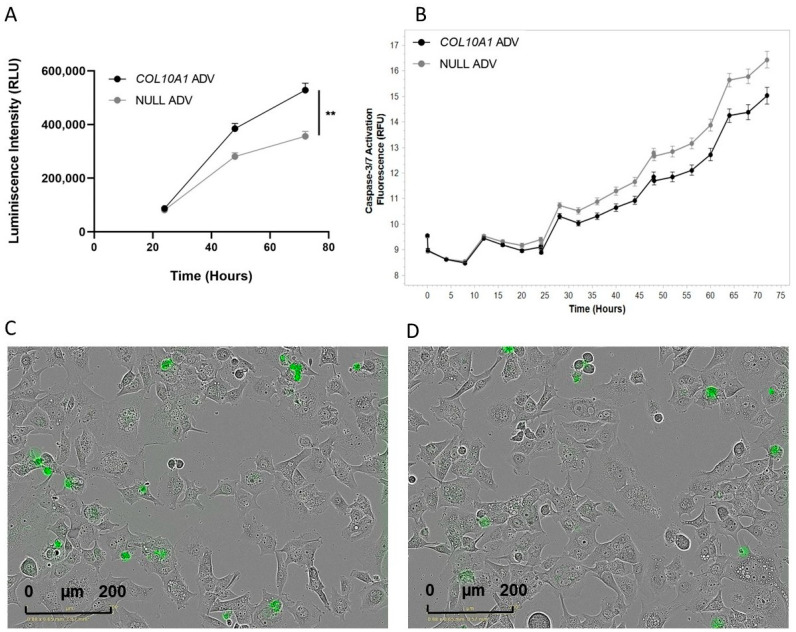
Effects of *COL10A1* ADV treatment on Hs746T cell proliferation and apoptosis. (**A**) *COL10A1* ADV treatment enhances the proliferative capacity of Hs746T cells. (**B**) *COL10A1* ADV treatment reduces apoptosis in Hs746T cells, as indicated by decreased Caspase-3/7 activation compared with untreated controls. (**C**) Representative fluorescence microscopy images showing apoptotic Hs746T cells infected with NULL ADV. (**D**) Representative fluorescence microscopy images showing apoptotic Hs746T cells infected with *COL10A1* ADV. ** (*p* < 0.01). (scale bar: 200 µm).

The upregulation of *COL10A1* gene expression increased the proliferation capacity of the Hs746T GC cell line when compared with control cells. Using an MTS-based assay, a significant increase in cell proliferation capacity (48%) was noted mainly at 72 h post-transduction (*p* = 0.004) (Figure 3A). Consistent with the increased proliferation, a decrease in apoptosis in Hs746T cells treated with *COL10A1* ADV compared with untreated cells at 72 h post-transduction was observed using the Incucyte^®^ Live-Cell Analysis device (Sartorius, Göttingen, Germany) (Figure 3B).

### 2.4. COL10A1 Overexpression Increases Gastric Cancer Cells Migration and Invasion via EMT

**Figure 4:**
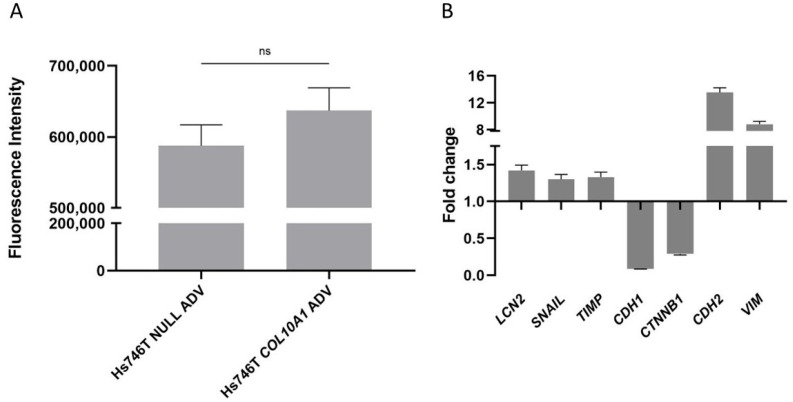
(**A**) Increased invasive capacity of Hs746T cells following *COL10A1* ADV treatments, 72h post-transduction. Mean fluorescence intensity for *COL10A1* ADV cells was positively correlated with invaded cell number, in contrast to control cells treated with NULL ADV. (**B**) Fold change in relative expression of EMT marker genes in Hs746T cells treated with *COL10A1* ADV versus NULL ADV, both normalized to *GAPDH*, at 48 h after transduction. ns means Not Significant.

The impact of *COL10A1* overexpression on cell migration and invasion was evaluated using the QCM ECMatrix Cell Invasion kit (Thermo Fisher Scientific, Waltham, MA, USA), and the results showed an increase in invasiveness by 8% in the case of Hs746T GC cells treated with *COL10A1* ADV at 72 h after transduction compared with control cells (Figure 4A). To further assess the impact of *COL10A1* overexpression on cell migration and invasion, we also analyzed the expression of the main genes involved in the EMT process, *E-cadherin*, *N-cadherin*, *Snail + Slug*, and *Vimentin* as important factors involved in promoting metastasis, using the RT-PCR technique. The results obtained showed that in Hs746T cells treated with *COL10A1* ADV, at 48 h post-transduction, a significant increase was observed in the expression of the *SNAIL*, *CDH2 (N-cadherin)*, *TIMP*, and *Vimentin* genes, genes that are markers for mesenchymal cells. A concomitant decrease in the expression of *CDH1* (*E-cadherin*) and *CTNNB1* (*β-catenin*) genes, markers for epithelial cells, was also observed (Figure 4B). These results indicate that COL10A1 promotes EMT and enhances the migratory and invasive potential of GC cells.

### 2.5. Analysis of the Effects of COL10A1 Overexpression on Intracellular Signaling Pathways

**Figure 5:**
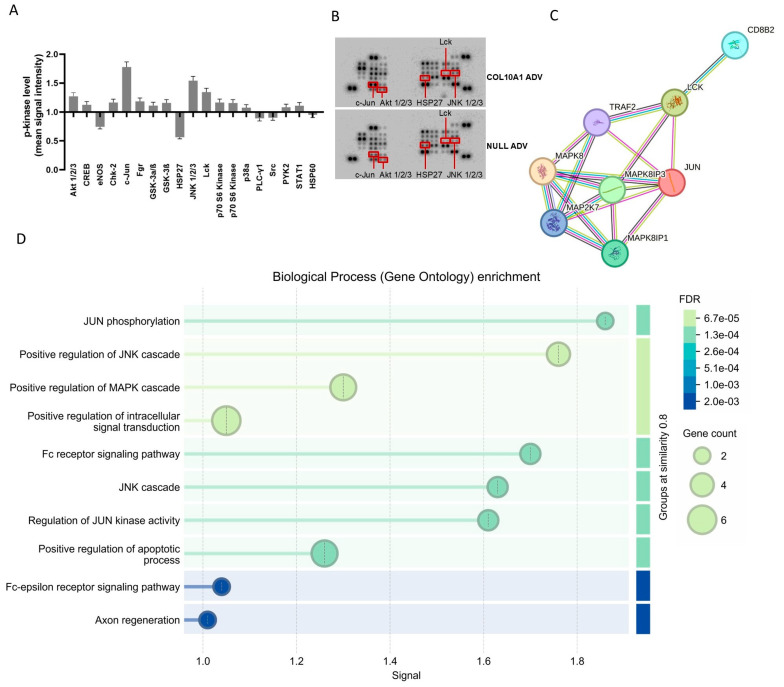
Changes in the expression of key signaling proteins in the gastric tumor cell line Hs746T following *COL10A1* ADV treatment (48 h post-transduction). (**A**) Quantitative analysis of relative protein expression levels in signaling pathways involved in gastric carcinogenesis, represented as fold changes compared to control (NULL ADV). (**B**) Protein array blots showing differential expression patterns between *COL10A1* ADV and NULL ADV-treated cells. (**C**) Protein–protein interaction (PPI) network of significantly altered proteins, highlighting their functional associations. (**D**) Gene Ontology (GO) enrichment analysis of biological processes regulated by the differentially expressed proteins, with dot size representing gene count and color indicating false discovery rate (FDR).

To investigate the intracellular mechanisms underlying COL10A1-mediated tumor progression, phosphokinase profiling was performed on Hs746T GC cells infected with *COL10A1* ADV and compared with control cells. The results revealed significant alterations in the phosphorylation of several proteins (Figure 5A,B). *COL10A1* overexpression led to increased phosphorylation and activation of Akt1/2/3, c-Jun, JNK1/2/3, and Lck, while a reduction was observed for eNOS and HSP27. The PPI enrichment analysis showed a high interaction between the activated kinases (STRING *p* = 0.000427) (Figure 5C,D). The reports showed functional enrichments and positive regulation of the JNK and MAPK cascade, pathways known to drive cell proliferation, survival, and tumorigenic potential.

## 3. Discussion

GC is a heterogeneous disease resulting from the accumulation of numerous genetic and epigenetic alterations, leading to the dysregulation of oncogenic and/or tumor suppressor signaling pathways. Diagnosis in the early stages of the tumor is hindered by the lack of circulating biomarkers with high sensitivity and specificity, and the fact that standard GC diagnosis relies mainly on invasive procedures, such as upper digestive endoscopy. In this context, the development of minimally or non-invasive diagnostic tests, as well as the identification of new biomarkers for GC with high specificity and sensitivity, remains essential. Also, the identification and validation of therapeutic targets could eliminate a large part of the side effects of currently used drugs. In previous studies, our team identified significant increases in the expression of several genes, including *COL10A1*, in gastric tumors compared to adjacent normal tissues. *COL10A1* has also been identified as a potential biomarker for early diagnosis of GC, as its increased expression occurs in early tumor stages and remains elevated during cancer progression [11]. Moreover, in another study, we showed that high plasma levels of COL10A1 are associated with advanced tumor stage in GC patients, and the elevated expression occurs from the beginning of carcinogenesis, in the early stages, and its increased level remains elevated during cancer progression [12]. These results are consistent with other studies that identified an overexpression of *COL10A1* in urothelial bladder, breast, colorectal, and pancreatic cancers, associated with tumor progression and poor prognosis [13,14,15,16]. Using high-throughput mRNA sequencing (RNA-seq), Tingting Li et al. identified *COL10A1* as the gene with the second highest expression level in both stage I and stage IV of GC [17]. Therefore, in our study, we used the TCGA database to evaluate the expression of *COL10A1* in gastric tumor tissue compared with normal adjacent tissue, and we also analyzed the *COL10A1* prognosis value in GC patients. The analysis showed that *COL10A1* is up-regulated in gastric tumor tissue, and an increased expression is associated with poor prognosis.

Recent studies, using single-cell RNA sequencing (scRNA-seq) analyses, have revealed that *COL10A1* is predominantly overexpressed in the tumor stroma of several solid cancers, including breast, pancreatic, and gastrointestinal tumors. Its expression is restricted to matrix-producing cancer-associated fibroblasts (CAFs), rather than cancer cells, and these COL10A1-positive CAFs display immunosuppressive and pro-metastatic properties. In basal cell carcinoma (BCC), scRNA-seq data from two independent datasets identified COL10A1-expressing stromal cells adjacent to infiltrative tumor regions, characterized by extracellular matrix remodeling features [18]. Similarly, in colorectal cancer, a COL10A1-positive fibroblast subpopulation (COL10A1^+^Fib) has been associated with tumor progression and poor prognosis in patients [19]. Moreover, in epithelial cancer cells, COL10A1 secreted by CAFs promotes EMT, thereby enhancing migration and invasion, and also induces M2 macrophage polarization, contributing to an immunosuppressive microenvironment [19]. Therefore, we chose to test in vitro the behavior of GC cells in the presence of an external source of COL10A1.

Accordingly, we performed in vitro experiments demonstrating that an increase in *COL10A1* expression decreases apoptosis and promotes proliferation, migration, and invasion by activating the expression of several genes involved in EMT, such as *SNAIL*, *CDH2* (*N-cadherin*), *TIMP*, and *Vimentin*. Although significant changes in EMT markers were observed, functional assays of migration and invasion did not reach statistical significance. This may reflect partial EMT states, temporal differences between molecular changes and phenotypic manifestation, or assay sensitivity. Therefore, molecular EMT changes may precede or occur independently of measurable changes in cell motility and invasiveness.

Data available from the literature indicate that *COL10A1* overexpression can affect the balance between tumor cell proliferation and apoptosis, thus sustaining the carcinogenesis process in different types of cancer. Thus, *COL10A1* upregulation promotes cell proliferation and metastasis and inhibits apoptosis and autophagy in lung cancer cells [4]. In cervical cancer cells, *COL10A1* upregulation promotes proliferation, migration, and EMT processes through activation of *N-cadherin* and *Vimentin* via TGF-β/Smad signaling [20]. *COL10A1* overexpression can also influence immunotherapy response and resistance to radiotherapy and chemotherapy in prostate cancer patients through mechanisms involving endoplasmic reticulum stress [21].

Our findings show that *COL10A1* overexpression also induced a significant increase in phosphorylation/activation levels of c-Jun, JNK, and 1/2/3Akt, proteins actively involved in cell proliferation, and a decrease in the level of phosphorylation/activation in the case of eNOS and HSP27 proteins. c-Jun phosphorylation, together with significant enrichment of the MAPK cascade, indicates activation of a pro-mitogenic signaling module that drives cell cycle gene expression via MAPK→AP-1. In parallel, reduced phosphorylation of HSP27—normally a regulator of actin dynamics, stress tolerance, and chaperone function—suggests cytoskeletal remodeling that favors proliferation and weakens stress defenses. This pattern is consistent with a signaling shift toward dominant ERK activity (with relative suppression of p38/MK2) or increased phosphatase activity, explaining the concurrent increase in p-c-Jun and decrease in p-HSP27. In recent years, JNK has been increasingly recognized as an attractive molecular target for cancer treatment due to its involvement in the regulation of cellular processes associated with carcinogenesis, including proliferation, differentiation and survival of tumor cells. JNK seems to sustain EMT and enhance GC cells invasion and migration [22]. c-Jun is a transcription factor with oncogenic function activated by the Jun N-terminal kinase (JNK), with a central role in cellular signal transduction, positively regulating cell proliferation by inhibiting the expression and function of tumor suppressor genes. c-Jun can also sustain the transcription of genes associated with ECM components and facilitate cell growth and invasion in cancer [23]. The phosphatidylinositol 3-kinase (PI3K)/protein kinase B (AKT)/mammalian target of rapamycin (mTOR) signaling pathway is involved in multiple cellular processes, including cell survival, proliferation, differentiation, metabolism, and cytoskeletal reorganization. Frequent activation of AKT has been reported in approximately 78% of GC cases and is associated with a poor prognosis in patients diagnosed with this type of cancer [24]. Thus, activation of these signaling pathways in Hs746T cells treated with *COL10A1* ADV is at the basis of the increased proliferative capacity of cells in which the *COL10A1* gene was overexpressed. Although our results provide novel insights into the role of COL10A1 in GC, further validation in additional GC cell lines, including AGS and NCI-N87, as well as GC–CAF co-culture systems, is necessary to strengthen the conclusions and confirm their broader relevance. Future studies should also include pathway inhibition experiments to determine whether the effects of COL10A1 are mediated directly through these signaling pathways.

## 4. Materials and Methods

### 4.1. Analysis of Databases

The expression levels of *COL10A1* gene in different types of solid tumors and pathological stages of GC were assessed with UALCAN web tool (http://ualcan.path.uab.edu/, accessed on 26 September 2025), based on TCGA online available RNAseq data [25,26]. Differential mRNA expression analysis includes normal tissues and all TNM stages of GC. The GEPIA web tool (http://gepia.cancer-pku.cn/, accessed on 26 September 2025) was used to show differences in gene expression between tumor and normal tissues using RNA sequencing data from the TCGA and GTEx databases [27]. The prognostic significance of *COL10A1* in GC was analyzed using the Kaplan–Meier Plotter web tool (https://kmplot.com/analysis/, accessed on 26 September 2025). The median level of *COL10A1* was utilized to categorize 875 patients with GC into high and low *COL10A1* groups. The two patient cohorts are compared by a Kaplan–Meier survival plot, and the hazard ratio with 95% confidence intervals and log-rank *p* value are calculated [28]. The *p*-value for Student’s *t*-test was set as follows: * *p* < 0.05, ** *p* < 0.01,*** *p* < 0.001.

### 4.2. Cell Culture

Human gastric adenocarcinoma cell lines AGS (catalog no. CRL-1739), NCI-N87 (catalog no. CRL-5822), and Hs746T (catalog no. HTB-135) were purchased from the American Type Culture Collection (Manassas, VA, USA). AGS cells were cultured in Ham’s F12 medium (Sigma Aldrich, St. Louis, MO, USA) supplemented with 10% fetal bovine serum (Biochrom, Cambridge, UK) at 37 °C in 5% CO_2_. The NCI-N87 cells were cultured in RPMI 1640 medium (Sigma Aldrich), and the Hs746T cells were cultured in Dulbecco’s Modified Eagle’s Medium (Sigma Aldrich), under the same culture conditions.

### 4.3. Cell Transduction

For the experiments of *COL10A1* gain of function in GC cell lines, a viral vector-based system was used (adenovirus) containing the *COL10A1* gene sequence (COL10A1 ADV) (Applied Biological Materials (abm) Inc., Richmond, BC, Canada), which has the ability to transiently induce the overexpression of the *COL10A1* gene. The recombinant adenoviral vector used is based on a replication-incompetent human adenovirus serotype 5 (Ad5) system lacking the *E1* and *E3* regions but retaining essential packaging signals and inverted terminal repeats. The absence of *E1* prevents viral replication and cell cycle progression, while deletion of *E3* reduces host immune suppression. Virus production and amplification were carried out in cells that express *E1* gene products (such as HEK293 cells). Thus, *COL10A1* ADV was amplified in HEK 293 cells, plated at 60–70% confluency. When more than 95% of HEK 293 cells were detached from the dishes, the cells and medium were collected, freeze-thawed three times, and centrifuged at 3000 rpm at room temperature for 10 min. The supernatant was collected and used for cell transduction as *COL10A1* ADV stock. Cell transduction was performed in Hs746T cell line and *COL10A1* ADV stock, and serial decimal dilutions from 10^−1^ to 10^−5^ were tested and evaluated at 24, 48, and 72 h intervals to identify the optimal concentration and the time required for transduction. Gene transduction was performed according to the producer protocol, and the transduction efficiency was evaluated by qPCR.

### 4.4. Quantitative Real-Time PCR Analysis

Evaluation of *COL10A1* gene expression was performed by the qRT-PCR technique. Cells were harvested 48 h after transduction, and total RNA was extracted with Tri Reagent (Sigma Aldrich). High Capacity cDNA Reverse Transcription Kit (Applied Biosystems, Waltham, MA, USA) was used for cDNA synthesis starting from 2 μg RNA while the quantitative RT-PCR reactions were performed on ABI 7300 Real-Time PCR System (Applied Biosystems) using pre-validated Taqman Gene Expression Assays kits (Applied Biosystems) and Maxima SYBR Green/ROX qPCR Master Mix for gene-specific primers (Thermo Fisher Scientific, Waltham, MA, USA). The primers used are obtained from OriGene Technologies Inc. (Rockville, MD, USA), and the primer sequences are described in Table 1. For *COL10A1*, we used the primer sequences described by Tingting Li et al. [17]. The PCR conditions were set as follows: 95 °C for 10 min; (95 °C for 15 s; 60 °C for 1 min), with 40 cycles and the results were analyzed with RQ study software (7300 System SDS v1.4, Applied Biosystems). The data were normalized to the housekeeping gene *GAPDH* transcripts and the comparative CT calculation (2^−ΔΔCt^ method) was employed to obtain relative expression of transcripts, with each reaction performed in triplicate.

### 4.5. Cell Proliferation

The cells were seeded in 96-well culture plates at a density of 6 × 10^3^ cells/well, divided into 2 groups: *COL10A1* ADV and NULL ADV. CellTiter 96 Aqueous One Solution (Promega, Madison, WI, USA) assay was used to evaluate the gene overexpression effect on cell proliferation at 48, 72, and 96 h after transduction. For each well, 20 μL MTS was added, and the plates were maintained for 4 h at 37 °C. The absorbance was measured at 550 nm using the TECAN GENios reader (Tecan Trading AG, Männedorf, Switzerland). All experiments were conducted in triplicate. Values were calculated as the percentage change induced by *COL10A1* ADV as compared to control (mock) cells.

### 4.6. Apoptosis

Apoptosis analysis in genetically modified cells was performed by analyzing the expression level of Caspase 3/7, using the Incucyte^®^ Live-Cell Analysis device (Sartorius, Göttingen, Germany). For this purpose, we used CellEvent Caspase-3/7 Green ReadyProbes Reagent (Thermo Fisher Scientific) for real-time discrimination in culture between live and dead cells. For this analysis, 3500 cells/well were seeded in a 96-well plate, the experiment being performed in triplicate. 24 h after seeding, the treatment with *COL10A1* ADV was performed on Hs746T cells, and the results were monitored in real time, comparing the behavior of infected cells with control (mock) cells.

### 4.7. Cell Migration and Invasion

In vitro testing of invasiveness based on the ability of tumor cells to modify extracellular matrix components was performed using the QCM ECMatrix Cell Invasion kit (Thermo Fisher Scientific). For this analysis, 3500 Hs746T cells/well were seeded in a 96-well plate. 48 h after the treatment with *COL10A1* ADV cells were starved for 6 h, trypsinized, and resuspended at a density of 3 × 10^5^ cells/well in serum-free medium in the 8.0 micron pore dishes inserted in a 24-well plate. In the lower part of the well, a medium containing 10% fetal bovine serum was added, and the system was maintained for 24 h at 37 °C (5% CO_2_). The cells that had migrated to the lower part of the insert were enzymatically detached, lysed, and stained for 15 min with DNA-binding dye, CyQuant GR (Thermo Fisher Scientific), and the results were obtained by reading the fluorescence at 480/520 nm (excitation/emission) using a Wallac Victor 2 spectrophotometer (Perkin Elmer, Waltham, MA, USA).

### 4.8. Protein Profiler Analysis

Analysis of the main signaling pathways involved in gastric carcinogenesis was performed using the dot-blot technique Human Phospho-Kinase Array Kit (Proteome ProfilerTM Array, R&D Systems, Minneapolis, MN, USA), which allows the simultaneous analysis of the phosphorylation profiles of the main phosphokinases and their protein substrates, proteins that are involved in signaling pathways that are usually altered in tumor cells. The protocol was performed according to the manufacturer’s protocol. For immunoblot analysis, 1 × 10^6^ cells grown in 6-well plates were transduced with *COL10A1* ADV or NULL ADV, and harvested in a lysis protein buffer after 48 h. 400 μg of total proteins diluted in the array buffer were incubated on the nitrocellulose membranes at 4 °C overnight. In the next step, the membranes were washed to remove unbound proteins and were incubated with a cocktail of biotinylated antibodies for 4 h. The final step has involved the repeated washing of the arrays and incubation with a streptavidin-HRP solution. The signals were detected using the chemiluminescent reaction, captured using MicroChemi 4.2 (Bio Imaging Systems, Jackson, MI, USA), and the images were analyzed using ImageJ 1.42 software (National Institute of Health, Bethesda, MD, USA) after subtraction of background levels (negative control) from sample signal levels and normalization to positive control signal to allow comparison between samples. Experiments were performed twice. Protein–Protein Interaction (PPI) Network Analysis was performed using STRING (https://string-db.org/, accessed on 1 October 2025) tool [29].

### 4.9. Statistical Analysis

All data were analyzed using GraphPad Prism 5.0. Results are presented as mean ± SD. Statistical significance was determined using Student’s unpaired *t*-test or one-/two-way ANOVA followed by the appropriate post hoc tests: Tukey for multiple comparisons between GC cell lines, Dunnett for comparing *COL10A1* ADV treatments with the NULL ADV control, and Bonferroni for proliferative assays. Differences in cell invasiveness were evaluated using an unpaired *t*-test. A *p*-value < 0.05 was considered statistically significant; * (*p* < 0.05), ** (*p* < 0.01), *** (*p* < 0.001), **** (*p* < 0.0001).

## 5. Conclusions

COL10A1 is markedly overexpressed in GC, with elevated levels that are associated with poor overall survival. Our findings reveal that COL10A1 acts as a functional driver of GC progression by enhancing key oncogenic processes, including proliferation, migration, invasion, and EMT. Moreover, the activation of major oncogenic pathways, such as PI3K/Akt/mTOR and JNK/MAPK signaling cascades, further supports its role in promoting tumor growth and survival. Together, these results highlight COL10A1 as a key element of the tumor microenvironment that contributes to aggressive GC phenotypes through remodeling of cellular signaling and EMT regulation. Overall, our results highlight COL10A1 as a promising molecular marker and potential therapeutic target, warranting additional studies to elucidate and confirm its role in promoting cell migration and invasion.

## Figures and Tables

**Table 1:** PCR Primer Sequences.

Gene	Primer Sequence	
*COL10A1*	F: 5′-AAGAATGGCACCCCTGTAATGT-3′	R: 5′-ACTCCCTGAAGCCTGATCCA-3′
*TIE*	F: 5′-GGTCAAGCAACCCAGCCTTTTC-3′	R: 5′-CAGGTCATTCCAGCAGAGCCAA-3′
*TIMP*	F: 5′-GGAGAGTGTCTGCGGATACTTC-3′	R: 5′-GCAGGTAGTGATGTGCAAGAGTC-3′
*CTNNB1*	F: 5′-CACAAGCAGAGTGCTGAAGGTG-3′	R: 5′-GATTCCTGAGAGTCCAAAGACAG-3′
*SNAIL*	F: 5′-TGCCCTCAAGATGCACATCCGA-3′	R: 5′-GGGACAGGAGAAGGGCTTCTC-3′
*CDH1*	F: 5′-GCCTCCTGAAAAGAGAGTGGAAG-3′	R: 5′-TGGCAGTGTCTCTCCAAATCCG-3′
*LCN2*	F: 5′-GTGAGCACCAACTACAACCAGC-3′	R: 5′-GTTCCGAAGTCAGCTCCTTGGT-3′
*CDH2*	F:5′-CCTCCAGAGTTTACTGCCATGAC-3	R: 5′-GTAGGATCTCCGCCACTGATTC-3′
*VIM*	F: 5′-AGGCAAAGCAGGAGTCCACTGA-3′	R: 5′-ATCTGGCGTTCCAGGGACTCAT-3′

## Data Availability

The original contributions presented in this study are included in the article. Further inquiries can be directed to the corresponding author.
